# Supplementary material for: NK cells are activated and primed for skin-homing during acute dengue virus infection in humans
Source: Nat Commun. 2019 Aug 29;10:3897. doi: 10.1038/s41467-019-11878-3 (PMC6715742; doi:10.1038/s41467-019-11878-3)
Supplement: Supplementary file 1 — Supplementary Information [file 41467_2019_11878_MOESM1_ESM.pdf]

Supplementary Materials

NK cells are activated and primed for skin-homing during acute dengue virus infection in humans

Christine L. Zimmer[1], Martin Cornillet[1], Carles Solà-Riera[1], Ka-Wai Cheung[2], Martin A. Ivarsson[1], Lim Mei Qiu[2], Nicole Marquardt[1], Yee Sin Leo[3,4,5,6], David Chie Lye[3,5,6], Jonas Klingström[1], Paul A. MacAry[7], Hans-Gustaf Ljunggren[1], Laura Rivino[2,8]*, and Niklas K. Björkström[1]*

*These authors contributed equally

Table of contents

Supplementary Figure 1

Supplementary Figure 2

Supplementary Figure 3

Supplementary Figure 4

Supplementary Figure 5

Supplementary Figure 6

Supplementary Figure 7

Supplementary Figure 8

Supplementary Table 1

Supplementary Table 2

Supplementary Table 3

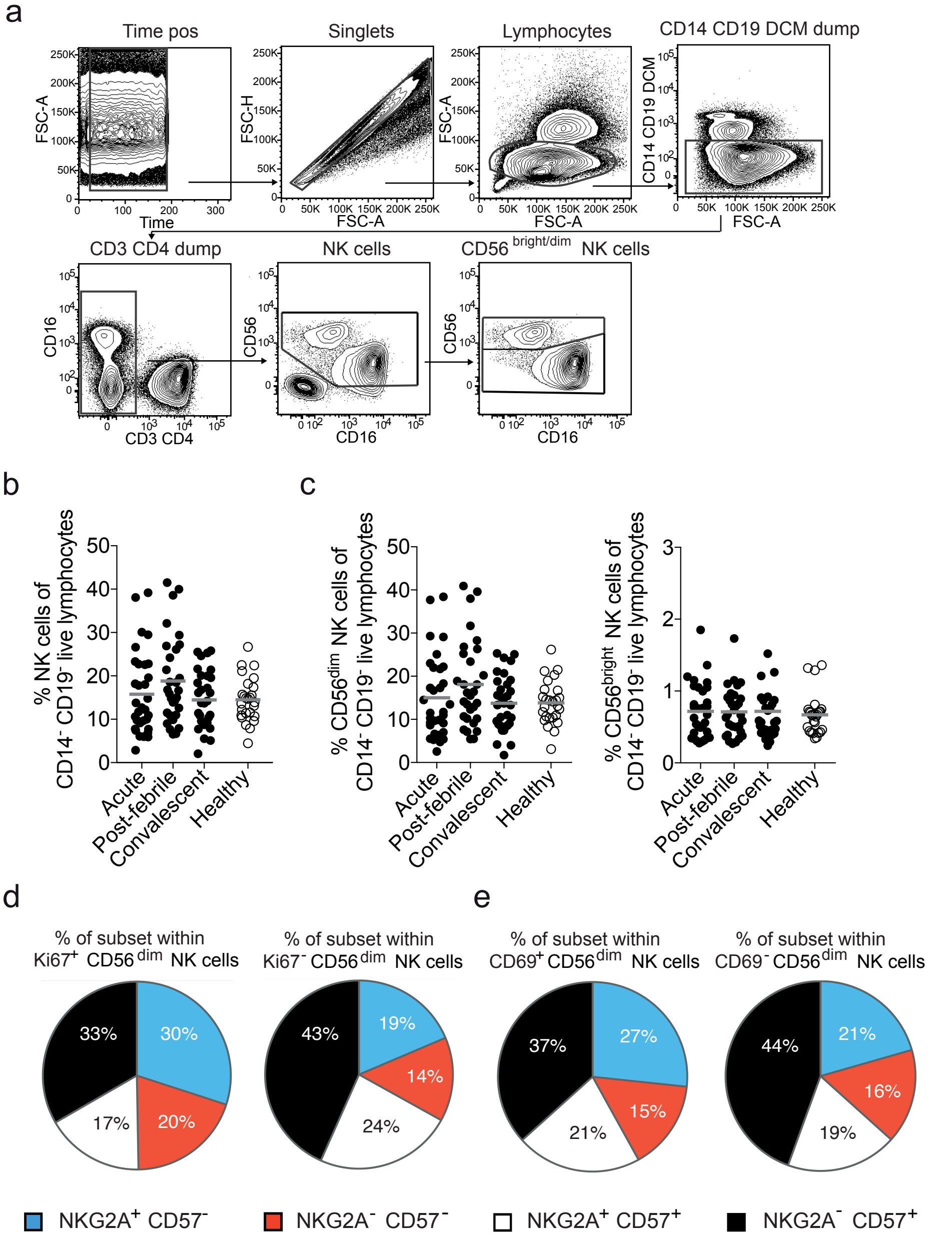

Supplementary Figure 1. Gating scheme and NK cell response during acute DENV infection. (**a**) Gating strategy to identify CD56^{bright} and CD56^{dim} NK cells from total PBMCs. A time-gate was applied first followed by gating on single cells, lymphocytes, dead cell marker-negative CD14^{-}CD19^{-} cells, CD3^{-}CD4^{-} cells, and finally on CD56^{+}CD16$^{+/-}$ cells before CD56^{bright} and CD56^{dim} NK cells were identified. (**b** and **c**) Summary of data for frequency of (**b**) total CD56^{+} NK cells, and (**c**) CD56^{bright} and CD56^{dim} NK cell subsets at the acute (n = 32), post-febrile (n = 30), and convalescent (n = 31) phases of DENV infection as compared to healthy controls (n = 26) with grey bars representing the mean. (**d** and **e**) Summary of results depicting frequencies of NKG2A^{+}CD57^{-}, NKG2A^{-}CD57^{-}, NKG2A^{+}CD57^{+}, and NKG2A^{-}CD57^{+} CD56^{dim} NK cells within responding (Ki67^{+} or CD69^{+}) or non-responding (Ki67^{-} or CD69^{-}) subsets of cells during the acute phase of infection (n = 25). Statistical differences were tested in (**b**) and (**c**) using one-way ANOVA and Kruskal-Wallis test followed by Tukey's multiple comparisons test or Dunn's multiple comparisons test, respectively. Source data are provided as a Source Data file.

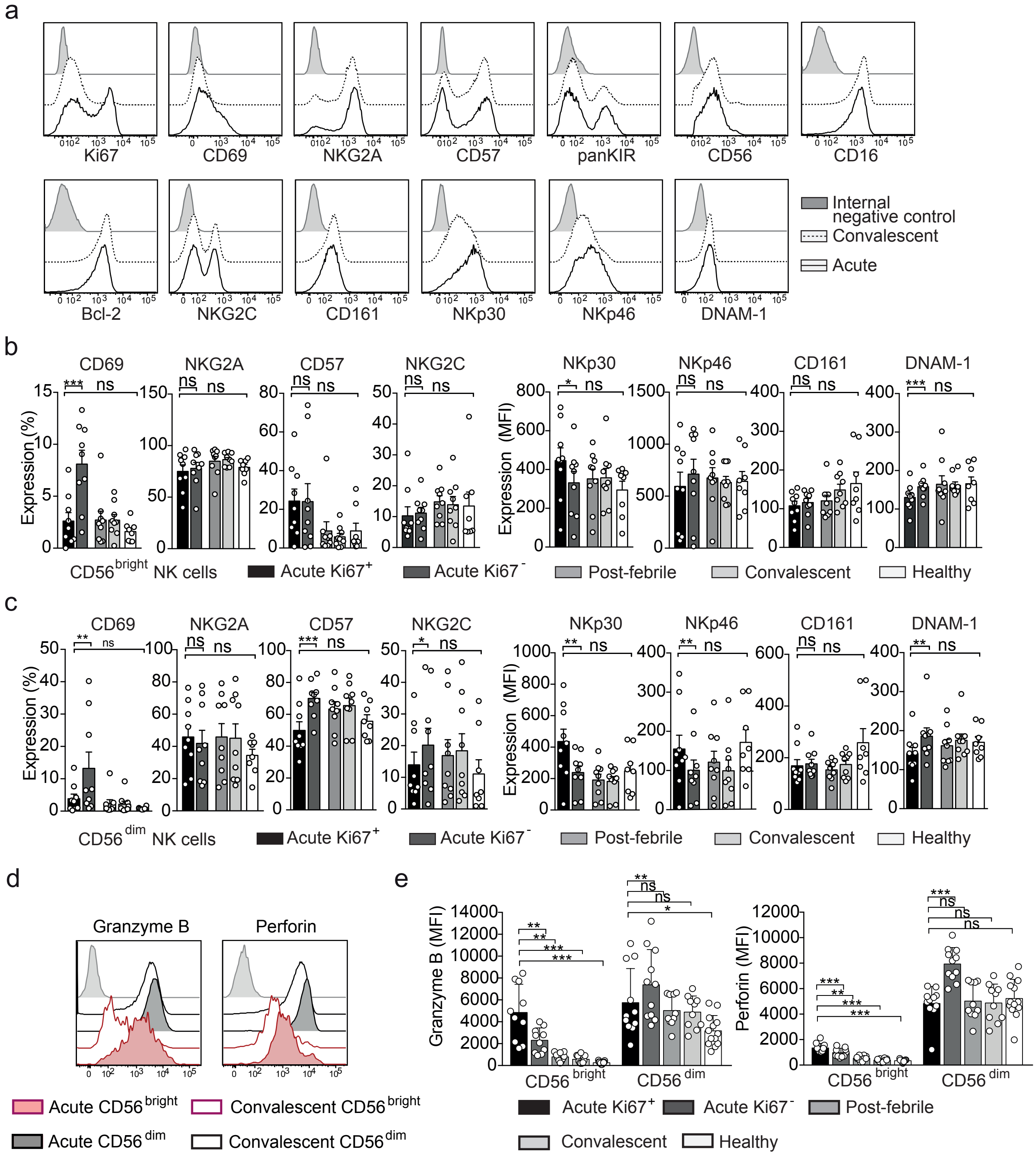

Supplementary Figure 2. Responding CD56^{dim} NK cells from DENV-infected patients display a less differentiated phenotype. (**a**) Representative histograms showing expression of the indicated markers on NK cells from one DENV-infected patient at the acute and convalescent phase of infection. Grey histograms in the background indicate internal negative control. (**b** and **c**) Summary of data showing expression (mean + SEM) of the indicated markers within responding and non-responding CD56^{bright} NK cells (**b**) and CD56^{dim} NK cells (**c**) during acute infection compared to post-febrile and convalescent phases (n = 9) as well as to healthy controls (n = 8). (**d**) Representative histograms showing granzyme B and perforin expression of CD56^{bright} NK cells (red) and CD56^{dim} NK cells (black) from the acute (filled) and convalescent (open) phase. (**e**) Summary graphs for granzyme B and perforin expression of CD56^{bright} and CD56^{dim} NK cells during the indicated stages of DENV infection (n = 10-11) and healthy controls (n = 13). Statistical differences were tested using paired and unpaired t-test or Wilcoxon matched-pairs signed rank test and Mann-Whitney test. * $p < 0.05$, ** $p < 0.01$, *** $p < 0.001$. Source data are provided as a Source Data file.

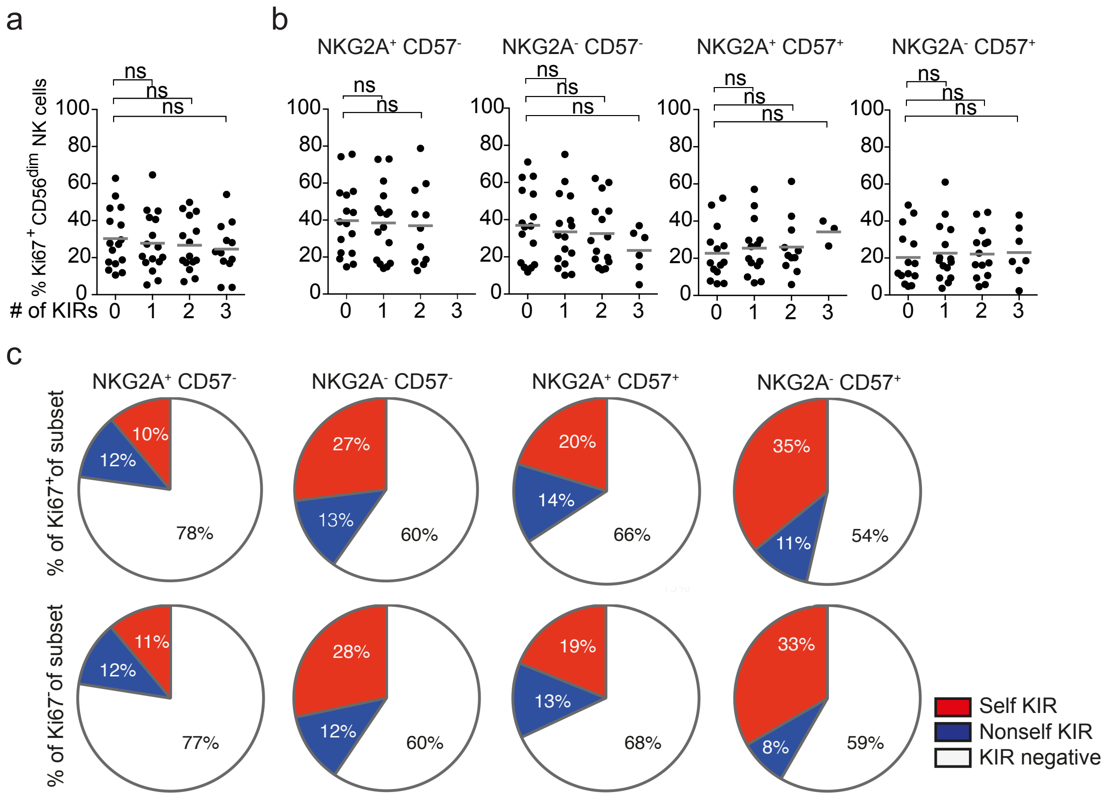

Supplementary Figure 3. CD56^{dim} NK cell responses are uncoupled from the numbers of KIRs expressed. (**a**) Boolean gates were used to identify CD56^{dim} NK cells expressing zero, one, two, or three KIRs. Only subsets with ≥ 50 events in the defining gate were analyzed. Summary data for frequency of Ki67 expression within CD56^{dim} NK cells expressing different numbers of KIRs is shown (n = 16). (**b**) Summary data for the frequency of Ki67-expression within NKG2A$^{+/-}$CD57$^{+/-}$ CD56^{dim} NK cell subsets expressing different numbers of KIRs (n = 16). (**c**) Frequency of KIR negative, self KIR^{+}, and non-self KIR^{+} CD56^{dim} NK cells within subsets defined by NKG2A and/or CD57 expression comparing responding (Ki67^{+}, top row) and non-responding (Ki67^{-}, bottom row) cells (n = 9). Statistical differences were tested using one-way ANOVA followed by Tukey's multiple comparison test. ns = not significant. Source data are provided as a Source Data file.

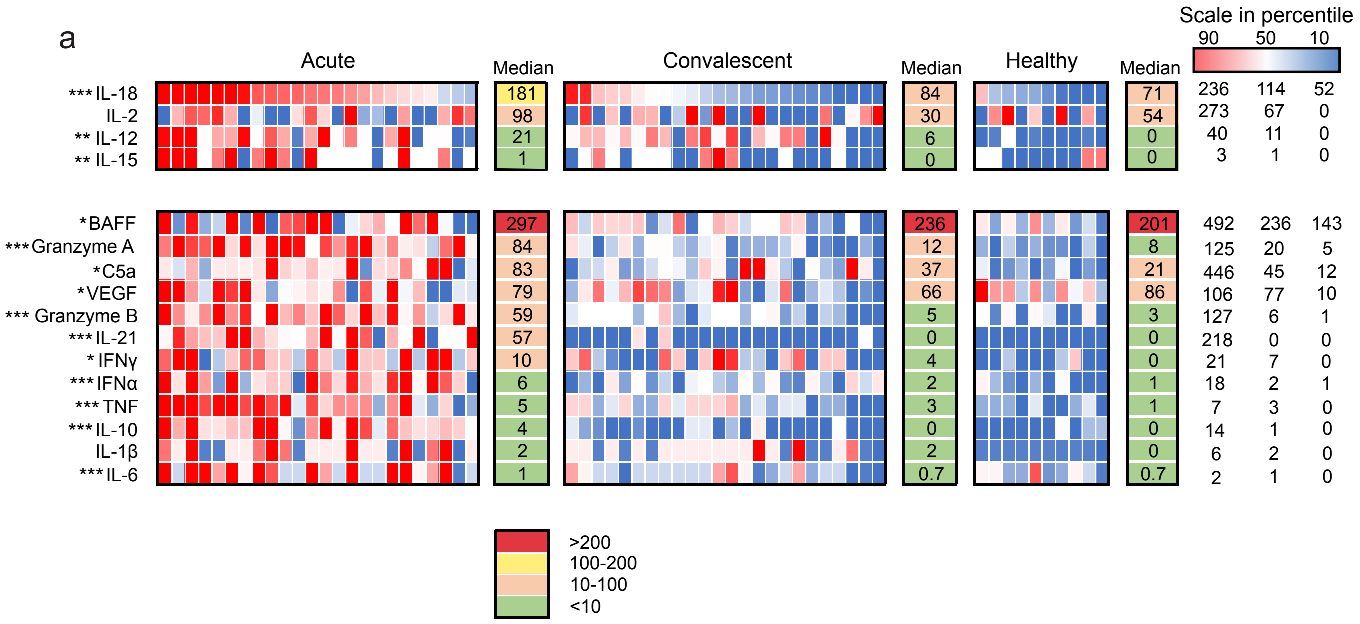

Supplementary Figure 4. Changes in plasma cytokine levels during acute DENV infection. **(a)** Heat map displaying expression pattern of 16 cytokines and soluble factors from plasma of DENV-infected patients at the acute and convalescent phase of infection (n = 24) as well as from healthy controls (n = 10). The color scale indicates the numbers within the 10th and 90th percentile within the range given for each parameter. IL-18, IL-12, IL-15, BAFF, granzyme A, granzyme B, TNF, IL-21, IL-10, C5a, VEGF, IFNγ, IFNα, and IL-6 were significantly increased at the acute phase as compared to the convalescent phase. Statistical differences were tested using Wilcoxon matched-pairs signed rank test. * $p < 0.05$, ** $p < 0.01$, *** $p < 0.001$. Source data are provided as a Source Data file.

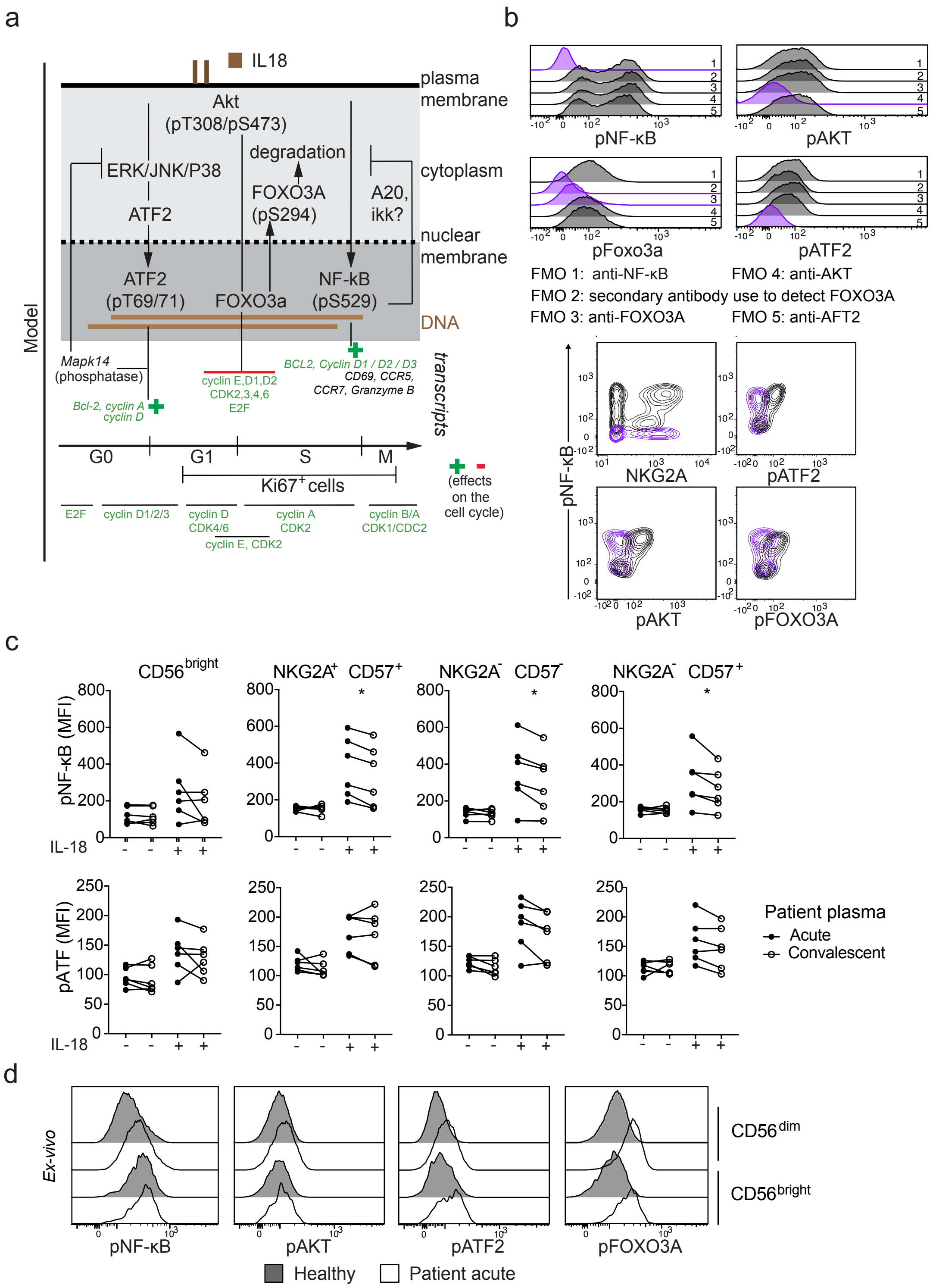

Supplementary Figure 5. Phospho-flow analysis of NK cell IL-18R-signaling. (**a**) Schematic illustration of signaling events downstream of the IL-18R. (**b**) Illustration of representative phospho-flow epitope staining using FMO controls. (**c**) Summary (MFI) of phospho-flow data for CD56^{bright} and NKG2A^{+}CD57^{+}, NKG2A^{-}CD57^{-}, and NKG2A^{-}CD57^{+} CD56^{dim} NK cell subsets (n = 6). (**d**) Representative histrograms showing ex-vivo pNF-κB, pAKT, pATF2, and pFOXO3A phospho-flow staining on CD56^{bright} and CD56^{dim} NK cells from the acute phase of DENV-infected patients and healthy controls. Statistical differences were tested using Wilcoxon matched-pairs signed rank test. * $p < 0.05$. Source data are provided as a Source Data file.

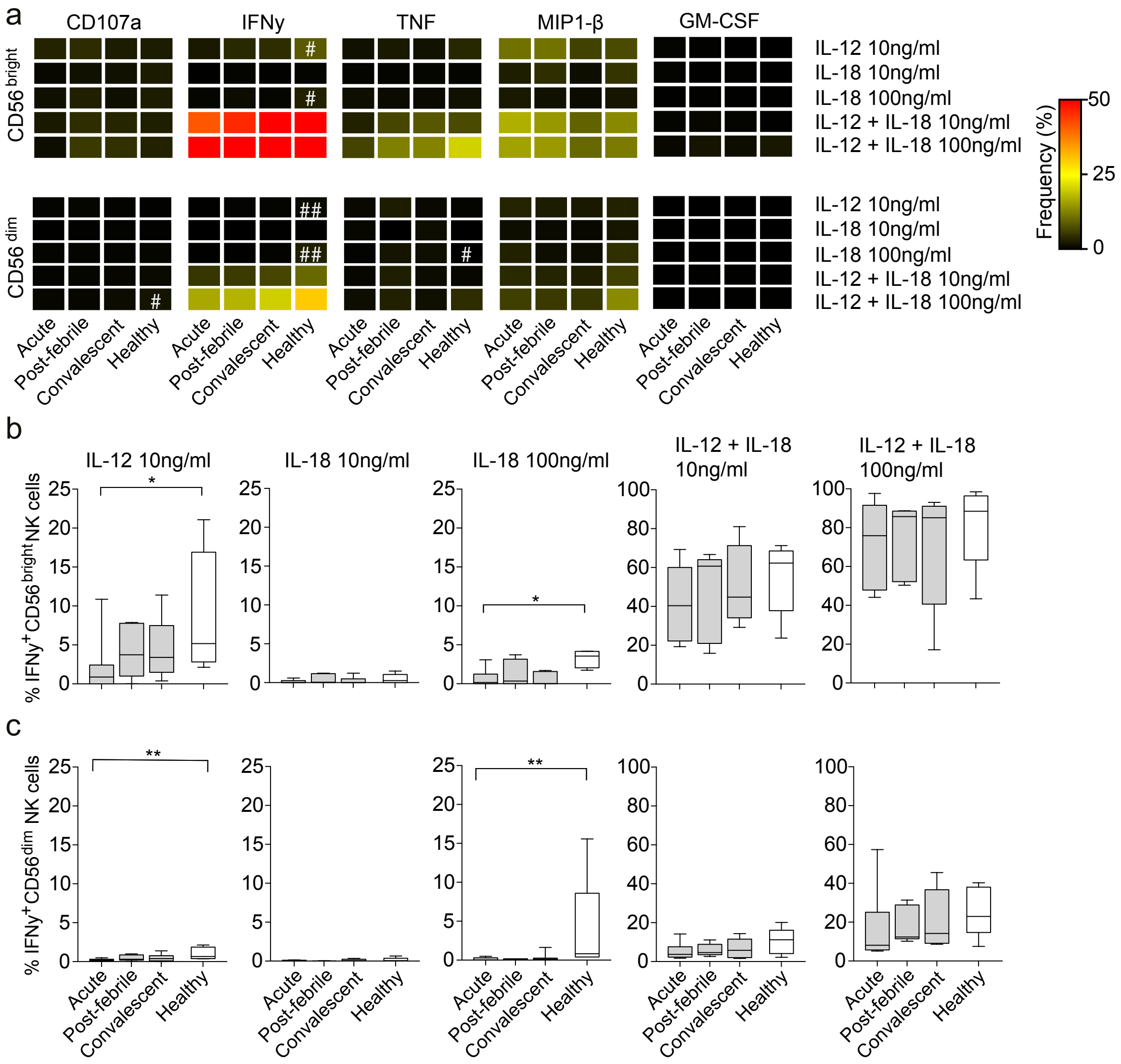

Supplementary Figure 6. Assessment of NK cell responsiveness to low-dose cytokine stimulation. (**a**) Heat map summarizing the frequency of $CD56^{bright}$ and $CD56^{dim}$ NK cell responses upon stimulation with the indicated combinations and concentrations of IL-12 and IL-18. NK cells were either from patients with different stages of DENV infection or healthy controls as indicated. (**b**) Frequency of IFNγ positive $CD56^{bright}$ (**c**) and $CD56^{dim}$ NK cells (n = 6-8). Lowest and highest observations are displayed with the median indicated as center line. Statistical differences were tested using Wilcoxon matched-pairs signed rank test or Mann-Whitney test. Hashes (#) in (**a**) represent significant differences comparing NK cells from patients with acute DENV infection to healthy controls; # $p < 0.05$, ## $p < 0.01$. Source data are provided as a Source Data file.

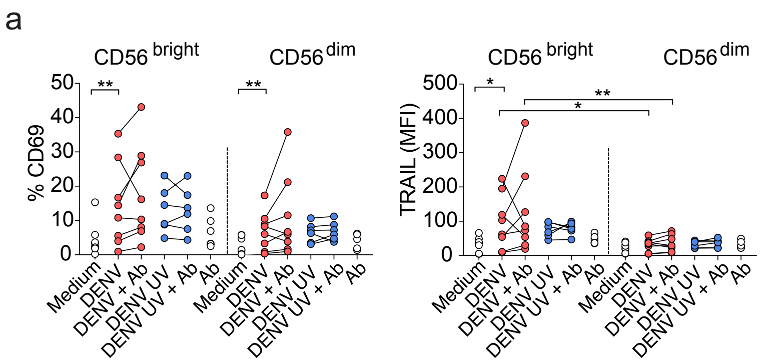

Supplementary Figure 7. **NK cell activation after in vitro infection of PBMC with DENV**. (**a**) Frequency of CD56^{bright} and CD56^{dim} NK cells expressing CD69 and TRAIL upon *in vitro* infection of healthy donor PBMCs with DENV, DENV pre-incubated with the 4G2 mAb, UV-inactivated DENV (DENV UV) with and without the 4G2 mAb, and with the 4G2 mAb only (Ab). Responses were measured 24h after infection. Statistical differences were tested using paired t-test or Wilcoxon matched-pairs signed rank test; * $p < 0.05$, ** $p < 0.01$. Source data are provided as a Source Data file.

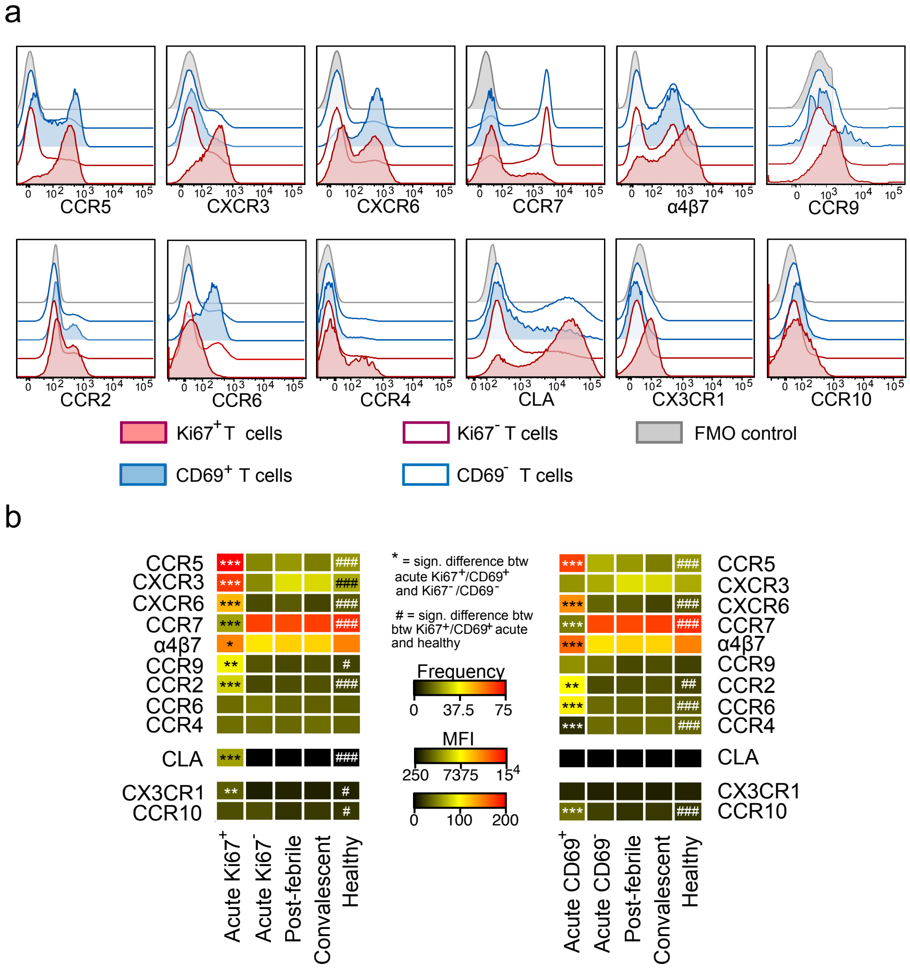

Supplementary Figure 8. Chemokine receptor profile on T cells during DENV infection. (**a**) Representative histograms showing the chemokine receptor expression on Ki67$^{+/-}$ (red) as well as CD69$^{+/-}$ (blue) T cells during the acute phase of DENV infection. (**b**) Heat map summarizing the median expression of 12 chemokine receptors on T cells subdivided into Ki67$^{+/-}$ cells (left panel) and CD69$^{+/-}$ cells (right panel) during the acute phase compared to the post-febrile and convalescent phase of DENV infection (n = 10-21) and healthy controls (n = 12-16). Statistical differences were tested using paired t-test or Wilcoxon matched-pairs signed rank test and unpaired t-test or Mann-Whitney test. Stars (*) represent Ki67^{+} and CD69^{+} compared to Ki67^{-} and CD69^{-}, respectively, * < 0.05, *** < 0.001. Hashes (#) represent Ki67^{+} or CD69^{+} compared to healthy controls, # $p < 0.05$, ## $p < 0.01$, ### $p < 0.001$. Source data are provided as a Source Data file.

Supplementary Table 1. Clinical characteristics of patients included in the study

Clinical parameters	Dengue patients
Subjects	32
Ethnicity	
Chinese	25/32 (78%)
Indian	4/32 (13%)
Others	3/32 (9%)
Age, median (range)	41 (23 - 63)
Gender	
Female	8/32 (25%)
Male	24/32 (75%)
Disease severity*	
DF	31/32 (97%)
DHF	1/32 (3%)
Skin manifestations†	15/32 (47%)
Gastrointestinal symptoms‡	15/32 (47%)
Liver symptoms§	6/32 (19%)
Lowest WBC count, median (range), 10^9/L	2.7 (1.2 - 8.2)
Highest hematocrit, median, (range), %	45.1 (38.7 - 51.8)
Lowest platelet count, median (range), 10^9/L	83 (19 - 198)
Highest ALT, median (range), U/L‖	49 (17 - 326)
Highest AST, median (range), U/L‖	68.5 (29 - 364)

*Cases were classified according to the revised WHO guidelines (*44*). DF dengue fever, DHF dengue hemorrhagic fever

†Skin manifestations include maculopapular skin rash and/or petechiae

‡Gut manifestations include vomiting, diarrhea and abdominal pain

§Liver manifestations include hepatomegaly and/or rise in liver enzymes of at least 2x upper limit of normal

‖ALT and AST levels were not available for 14 of the patient

Supplementary Table 2. KIR and KIR-ligand genotype of DF patients

Patient ID	KIR							Ligand		
Donor	KIR 2DL1	2DL2	2DL3	3DL1	2DS1	2DS2	2DS4	3DS1	C1	C2	Bw4
DENV-infected											
Mab 131	x	x	x	x		x	x		x		x
Mab 132	x		x	x	x		x	x	x		x
Mab 139	x	x	x		x	x	x	x	x		x
Mab 142	x		x	x			x		x	x	x
Mab 145		x		x	x	x				x	x
Mab 152			x	x	x			x	x	x	x
Mab 161	x		x	x	x			x	x	x	
Mab 168	x		x	x	x		x	x	x		x
Mab 172	x	x	x	x	x			x	x		x
Mab 203	x	x	x	x		x	x		x	x	x
Mab 224	x		x	x			x		x		x
Mab 228	x		x	x					x		x
Mab 291	x		x	x	x		x	x	x	x	x
Mab 323	x	x	x	x	x	x	x	x	x		x
Mab 324	x	x	x			x	x		x		
Mab 325	x		x	x			x		x		x

Supplementary Table 3. Antibodies used for flow cytometry

Antibody	Clone	Dilution	Catalogue number	Company
**Extracellular stainings**				
DNAM-1 FITC	DX11	1/20	559788	BD Bioscience
NKp30 Alexa Fluor 647	RUO	1/100	558408	BD Bioscience
NKp46 Brilliant Violet 421	9E2	1/50	564065	BD Bioscience
CD3 Alexa Fluor 700	UCHT1	1/100	557943	BD Bioscience
CD3 Brilliant Violet 510	UCHT1	1/25	563109	BD Bioscience
CD3 V450	UCHT1	1/20	560365	BD Bioscience
CD14 Horizon V500	M5E2	1/100	561391	BD Bioscience
CD16 V500	3G8	1/20	561393	BD Bioscience
CD16 Brilliant Violet 711	3G8	1/100	302044	BD Bioscience
CD19 Horizon V500	HIB19	1/100	561121	BD Bioscience
CD19 Brilliant Violet 510	SJ24C1	1/100	562947	BD Bioscience
CD56 Brilliant Violet 711	MAb11	1/100	563418	BD Bioscience
CD56 BUV737	NCAM16.2	1/100	564447	BD Bioscience
CD56 CF-594	NCAM16.2	1/100	564849	BD Bioscience
CD57 Brilliant Violet 605	NK-1	1/200	563895	BD Bioscience
CD69 BUV395	FN50	1/50	564364	BD Bioscience
CD69 BUV737	FN50	1/50	612817	BD Bioscience
CD69 APC-Cy7	FN50	1/50	557756	BD Bioscience
CD107a FITC	RUO	1/20	555800	BD Bioscience
KIR2DL2/L3/S2 BB515	CH-L	1/50	564678	BD Bioscience
KIR2DL2/L3/S2 BB515	CH-L	1/50	564678	BD Bioscience
CCR5 BUV395	2D7/CCR5	1/25	565224	BD Bioscience
CCR5 BUV737	2D7/CCR5	1/10	565293	BD Bioscience
CCR5 FITC	2D7/CCR5	1/10	555992	BD Bioscience
CCR7 Alexa Fluor 700	150503	1/20	561143	BD Bioscience
CD3 PE-Cy5	UCHT1	1/100	300410	BioLegend
CD4 PE-Cy5	OKT4	1/200	317412	BioLegend
CD16 Brilliant Violet 785	3G8	1/300	302045	BioLegend
CD16 Brilliant Violet 570	3G8	1/200	302036	BioLegend

CD38 Brilliant Violet 650	HB-7	1/50	356620	BioLegend
CD45 Alexa Fluor 700	HI30	1/400	304024	BioLegend
CD56 Brilliant Violet 711	HCD56	1/100	318336	BioLegend
CD57 Pacific Blue	HCD57	1/200	322316	BioLegend
CD69 Brilliant violet 785	FN50	1/50	310932	BioLegend
CD161 Brilliant Violet 605	HP-3G10	1/200	339916	BioLegend
CCR2 FITC	K036C2	1/10	357216	BioLegend
CCR4 PE-Cy7	L291H7	1/10	359410	BioLegend
CCR5 APC Cy7	J418F1	1/10	359110	BioLegend
CCR6 Brillaint Violet 711	G034E3	1/25	353436	BioLegend
CCR7 Brilliant Violet 421	G043H7	1/25	353208	BioLegend
CCR9 Alexa Fluor 647	L053E8	1/5	358912	BioLegend
CCR10 PE	6588-5	1/25	341504	BioLegend
CXCR3 PE-Cy7	G025H7	1/25	353720	BioLegend
CXCR6 AF 647	K041E5	1/50	356008	BioLegend
CXCR6 Brilliant Violet 421	K041E5	1/25	356014	BioLegend
CLA FITC	HECA-452	1/10	321306	BioLegend
CLA Pacific Blue	HECA-452	1/100	321308	BioLegend
CX3CR1 APC-Cy7	2A9-1	1/25	341616	BioLegend
NKp46 biotin	9E2	1/50	325106	BioLegend
KIR3DL1 Alexa Fluor 700	DX9	1/200	312712	BioLegend
IL-18Ra PE	H44	1/100	313808	BioLegend
HLA-DR APC Cy7	L243	1/25	307618	BioLegend
TRAIL APC	RIK-2	1/25	308210	BioLegend
CD57 purified	TB01	1/100	16057785	eBioscience
CCR10 APC	341305	1/5	FAB3478A	R&D Systems
NKG2C PE	FAB138P	1/25	FAB138P	R&D Systems
KIR2DL1/S1 PE-Cy5.5	143211	1/20	a66898	Beckman Coulter
NKG2A PE-Cy7	Z199	1/100	PNB10246	Beckman Coulter
NKG2A Alexa Fluor 647	Z199	1/25	A60797	Beckman Coulter
NKG2A PE	REA110	1/400	130113566	Miltenyi
NKG2A VioBright FITC	REA110	1/25	130105646	Miltenyi
NKG2A APC	REA110	1/100	130113563	Miltenyi
CD14 PerCP	TüK4	1/25	130113150	Miltenyi

CD14 FITC	TüK4	1/25	130080701	Miltenyi
CD57 APC-Vio77	TB03	1/50	130116503	Miltenyi
KIR2DL3 PE-Vio770	REA147	1/20	130100117	Miltenyi
KIR2DL1 APV-Vio770	11PB6	1/20	130103937	Miltenyi
KIR2DS4 biotin	JJC11.6	1/50	130092898	Miltenyi
a4b7 purified		1/50		NIH
**Secondary extracellular stainings**				
streptavidin-Qdot585		1/200	Q10111MP	Life technologies
steptavidin-PE-CF594		1/200	562318	BD Biosciences
anti-IgM eF650	R6-60.2	1/100	564027	BD Biosciences
Goat anti-Rabbit IgG (H+L) PE-Alexa Fluor 647		1/500	A20991	Thermo Fisher
**Intracellular stainings**				
Ki67 Alexa Fluor 700	B56	1/100	561277	BD Biosciences
TNF Brilliant Violet 650	Mab11	1/25	563418	BD Biosciences
IFN gamma Brilliant Violet 421	B27	1/200	562988	BD Biosciences
MIP-1b PE	D21-1351	1/50	550078	BD Biosciences
MIP-1b Alexa Fluor 700	D21-1351	1/50	D211351	BD Biosciences
GM-CSF PE-CF594	BVD2-21C11	1/100	562857	BD Biosciences
Granzyme B PE-CF594	GB11	1/50	562462	BD Biosciences
Bcl-2 PE-CF594	BCL2/100	1/100	563601	BD Biosciences
p-Akt (pT308) PE	J1-223.371	1/25	558275	BD Bioscience
p-Akt (pS473) PE	M89-61	1/25	560378	BD Bioscience
p-NF-κBp65(pS529) PE-CF594	K10-895.12.50	1/25	565447	BD Bioscience
Ki67 Brilliant Violet 421	Ki-67	1/100	350505	BioLegend
IFN gamma Brilliant Violet 785	4S.B3	1/200	502541	BioLegend
Perforin Brilliant Violet 421	Dg9	1/100	30122	BioLegend
Perforin PE Cy-7	B-D48	1/55	353315	BioLegend

p-ATF2(Thr69/71) Alexa Fluor 647	AW65	1/25	FCMAB271A6	Merk millipore
p-FOXO3A (Ser294)		1/25	5538S	Cell signaling
