# Supplementary material for: NK cells are activated and primed for skin-homing during acute dengue virus infection in humans
Source: Nat Commun. 2019 Aug 29;10:3897. doi: 10.1038/s41467-019-11878-3 (PMC6715742; doi:10.1038/s41467-019-11878-3)
Supplement: Supplementary file 3 — Reporting Summary [file 41467_2019_11878_MOESM3_ESM.pdf]

nature research

Corresponding author(s): Niklas K Björkström

Last updated by author(s): Jul 30, 2019

Reporting Summary

Nature Research wishes to improve the reproducibility of the work that we publish. This form provides structure for consistency and transparency in reporting. For further information on Nature Research policies, see Authors & Referees and the Editorial Policy Checklist.

Statistics

For all statistical analyses, confirm that the following items are present in the figure legend, table legend, main text, or Methods section.

n/a	Confirmed	
☐	☒	The exact sample size (*n*) for each experimental group/condition, given as a discrete number and unit of measurement
☐	☒	A statement on whether measurements were taken from distinct samples or whether the same sample was measured repeatedly
☐	☒	The statistical test(s) used AND whether they are one- or two-sided<br>*Only common tests should be described solely by name; describe more complex techniques in the Methods section.*
☒	☐	A description of all covariates tested
☐	☒	A description of any assumptions or corrections, such as tests of normality and adjustment for multiple comparisons
☐	☒	A full description of the statistical parameters including central tendency (e.g. means) or other basic estimates (e.g. regression coefficient) AND variation (e.g. standard deviation) or associated estimates of uncertainty (e.g. confidence intervals)
☐	☒	For null hypothesis testing, the test statistic (e.g. *F*, *t*, *r*) with confidence intervals, effect sizes, degrees of freedom and *P* value noted<br>*Give P values as exact values whenever suitable.*
☒	☐	For Bayesian analysis, information on the choice of priors and Markov chain Monte Carlo settings
☒	☐	For hierarchical and complex designs, identification of the appropriate level for tests and full reporting of outcomes
☐	☒	Estimates of effect sizes (e.g. Cohen's *d*, Pearson's *r*), indicating how they were calculated

Our web collection on statistics for biologists contains articles on many of the points above.

Software and code

Policy information about availability of computer code

Data collection	Patient material was collected as described in the manuscript. Samples were acquired on BD LSR Fortessa equipped with 5 lasers (BD Biosciences). Cytokines were measure using a BioPLex MAGPIX Multiplex Reader.
Data analysis	Flow cytometry data analysis was performed using FlowJo version 9.9.4 (TreeStar). Post-processing was performed using SPICE version 5.3 (provided by M. Roederer and J. Nozzi, NIAID, NIH) as well as R version 3.3.1 (The R Foundation of Statistical Computing)

For manuscripts utilizing custom algorithms or software that are central to the research but not yet described in published literature, software must be made available to editors/reviewers. We strongly encourage code deposition in a community repository (e.g. GitHub). See the Nature Research guidelines for submitting code & software for further information.

Data

Policy information about availability of data

All manuscripts must include a data availability statement. This statement should provide the following information, where applicable:

- Accession codes, unique identifiers, or web links for publicly available datasets
- A list of figures that have associated raw data
- A description of any restrictions on data availability

The authors declare that the data supporting the findings of this study are available in the article, the supplementary information files, in a source data file when stated, or upon request to the authors.

Field-specific reporting

Please select the one below that is the best fit for your research. If you are not sure, read the appropriate sections before making your selection.

☒ Life sciences ☐ Behavioural & social sciences ☐ Ecological, evolutionary & environmental sciences

For a reference copy of the document with all sections, see nature.com/documents/nr-reporting-summary-flat.pdf

Life sciences study design

All studies must disclose on these points even when the disclosure is negative.

Sample size	The sample size was n=31 to make sure that we have enough samples for running all experimental conditions with sufficient numbers that statistical calculations could be performed. For some experiment using healthy donors a minimum sample size of n=6 was chosen. For the collection of skin blister fluid, all patients that could possibly get recruited were included.
Data exclusions	In FACS analysis, data from patients were excluded when the mother population was below 50 cells and was therefore not possible to include in the analysis
Replication	2-3 patients (with two to three time points per patient) were run per experiments. In each experiments DENV patients and healthy controls were included to avoid bias. Data could be reproduced.
Randomization	In each experiments DENV patients and healthy controls were included to avoid an experimental bias for individual experiments. An internal control (always from the same buffy coat) was included in all experiments.
Blinding	No blinding was performed.

Reporting for specific materials, systems and methods

We require information from authors about some types of materials, experimental systems and methods used in many studies. Here, indicate whether each material, system or method listed is relevant to your study. If you are not sure if a list item applies to your research, read the appropriate section before selecting a response.

Materials & experimental systems

n/a	Involved in the study
☐	☒ Antibodies
☐	☒ Eukaryotic cell lines
☒	☐ Palaeontology
☒	☐ Animals and other organisms
☐	☒ Human research participants
☒	☐ Clinical data

Methods

n/a	Involved in the study
☒	☐ ChIP-seq
☐	☒ Flow cytometry
☒	☐ MRI-based neuroimaging

Antibodies

Antibodies used

Extracellular stainings
DNAM-1 FITC DX11 1/20 559788 BD Bioscience
NKp30 Alexa Fluor 647 RUO 1/100 558408 BD Bioscience
NKp46 Brilliant Violet 421 9E2 1/50 564065 BD Bioscience
CD3 Alexa Fluor 700 UCHT1 1/100 557943 BD Bioscience
CD3 Brilliant Violet 510 UCHT1 1/25 563109 BD Bioscience
CD3 V450 UCHT1 1/20 560365 BD Bioscience
CD14 Horizon V500 M5E2 1/100 561391 BD Bioscience
CD16 V500 3G8 1/20 561393 BD Bioscience
CD16 Brilliant Violet 711 3G8 1/100 302044 BD Bioscience
CD19 Horizon V500 HIB19 1/100 561121 BD Bioscience
CD19 Brilliant Violet 510 SJ24C1 1/100 562947 BD Bioscience
CD56 Brilliant Violet 711 MAb11 1/100 563418 BD Bioscience
CD56 BUV737 NCAM16.2 1/100 564447 BD Bioscience
CD56 CF-594 NCAM16.2 1/100 564849 BD Bioscience
CD57 Brilliant Violet 605 NK-1 1/200 563895 BD Bioscience
CD69 BUV395 FN50 1/50 564364 BD Bioscience
CD69 BUV737 FN50 1/50 612817 BD Bioscience
CD69 APC-Cy7 FN50 1/50 557756 BD Bioscience
CD107a FITC RUO 1/20 555800 BD Bioscience
KIR2DL2/L3/S2 BB515 CH-L 1/50 564678 BD Bioscience

KIR2DL2/L3/S2 BB515 CH-L 1/50 564678 BD Bioscience
CCR5 BUV395 2D7/CCR5 1/25 565224 BD Bioscience
CCR5 BUV737 2D7/CCR5 1/10 565293 BD Bioscience
CCR5 FITC 2D7/CCR5 1/10 555992 BD Bioscience
CCR7 Alexa Fluor 700 150503 1/20 561143 BD Bioscience
CD3 PE-Cy5 UCHT1 1/100 300410 BioLegend
CD4 PE-Cy5 OKT4 1/200 317412 BioLegend
CD16 Brilliant Violet 785 3G8 1/300 302045 BioLegend
CD16 Brilliant Violet 570 3G8 1/200 302036 BioLegend
CD38 Brilliant Violet 650 HB-7 1/50 356620 BioLegend
CD45 Alexa Fluor 700 HI30 1/400 304024 BioLegend
CD56 Brilliant Violet 711 HCD56 1/100 318336 BioLegend
CD57 Pacific Blue HCD57 1/200 322316 BioLegend
CD69 Brilliant violet 785 FN50 1/50 310932 BioLegend
CD161 Brilliant Violet 605 HP-3G10 1/200 339916 BioLegend
CCR2 FITC K036C2 1/10 357216 BioLegend
CCR4 PE-Cy7 L291H7 1/10 359410 BioLegend
CCR5 APC Cy7 J418F1 1/10 359110 BioLegend
CCR6 Brillaint Violet 711 G034E3 1/25 353436 BioLegend
CCR7 Brilliant Violet 421 G043H7 1/25 353208 BioLegend
CCR9 Alexa Fluor 647 L053E8 1/5 358912 BioLegend
CCR10 PE 6588-5 1/25 341504 BioLegend
CXCR3 PE-Cy7 G025H7 1/25 353720 BioLegend
CXCR6 AF 647 K041E5 1/50 356008 BioLegend
CXCR6 Brilliant Violet 421 K041E5 1/25 356014 BioLegend
CLA FITC HECA-452 1/10 321306 BioLegend
CLA Pacific Blue HECA-452 1/100 321308 BioLegend
CX3CR1 APC-Cy7 2A9-1 1/25 341616 BioLegend
NKp46 biotin 9E2 1/50 325106 BioLegend
KIR3DL1 Alexa Fluor 700 DX9 1/200 312712 BioLegend
IL-18Ra PE H44 1/100 313808 BioLegend
HLA-DR APC Cy7 L243 1/25 307618 BioLegend
TRAIL APC RIK-2 1/25 308210 BioLegend
CD57 purified TB01 1/100 16057785 eBioscience
CCR10 APC 341305 1/5 FAB3478A R&D Systems
NKG2C PE FAB138P 1/25 FAB138P R&D Systems
KIR2DL1/S1 PE-Cy5.5 143211 1/20 a66898 Beckman Coulter
NKG2A PE-Cy7 Z199 1/100 PNB10246 Beckman Coulter
NKG2A Alexa Fluor 647 Z199 1/25 A60797 Beckman Coulter
NKG2A PE REA110 1/400 130113566 Miltenyi
NKG2A VioBright FITC REA110 1/25 130105646 Miltenyi
NKG2A APC REA110 1/100 130113563 Miltenyi
CD14 PerCP TüK4 1/25 130113150 Miltenyi
CD14 FITC TüK4 1/25 130080701 Miltenyi
CD57 APC-Vio77 TB03 1/50 130116503 Miltenyi
KIR2DL3 PE-Vio770 REA147 1/20 130100117 Miltenyi
KIR2DL1 APV-Vio770 11PB6 1/20 130103937 Miltenyi
KIR2DS4 biotin JJC11.6 1/50 130092898 Miltenyi
a4b7 purified 1/50 NIH

Secondary extracellular stainings
streptavidin-Qdot585 1/200 Q10111MP Life technologies
steptavidin-PE-CF594 1/200 562318 BD Biosciences
anti-IgM eF650 R6-60.2 1/100 564027 BD Biosciences
Goat anti-Rabbit IgG (H+L) PE-Alexa Fluor 647 1/500 A20991 Thermo Fisher

Intracellular stainings
Ki67 Alexa Fluor 700 B56 1/100 561277 BD Biosciences
TNF Brilliant Violet 650 Mab11 1/25 563418 BD Biosciences
IFN gamma Brilliant Violet 421 B27 1/200 562988 BD Biosciences
MIP-1b PE D21-1351 1/50 550078 BD Biosciences
MIP-1b Alexa Fluor 700 D21-1351 1/50 D211351 BD Biosciences
GM-CSF PE-CF594 BVD2-21C11 1/100 562857 BD Biosciences
Granzyme B PE-CF594 GB11 1/50 562462 BD Biosciences
Bcl-2 PE-CF594 BCL2/100 1/100 563601 BD Biosciences
p-Akt (pT308) PE J1-223.371 1/25 558275 BD Bioscience
p-Akt (pS473) PE M89-61 1/25 560378 BD Bioscience
p-NF-κBp65(pS529) PE-CF594 K10-895.12.50 1/25 565447 BD Bioscience
Ki67 Brilliant Violet 421 Ki-67 1/100 350505 BioLegend
IFN gamma Brilliant Violet 785 4S.B3 1/200 502541 BioLegend
Perforin Brilliant Violet 421 Dg9 1/100 30122 BioLegend
Perforin PE Cy-7 B-D48 1/55 353315 BioLegend
p-ATF2(Thr69/71) Alexa Fluor 647 AW65 1/25 FCMAB271A6 Merk millipore
p-FOXO3A (Ser294) 1/25 5538S Cell signaling

Validation

All antibodies were validated by the manufacturers for flow cytometry as stated by the respective company

Eukaryotic cell lines

Policy information about cell lines

Cell line source(s)

ATCC K562 cells or 721.221 cells

Authentication

The cell cells are commercially available at ATCC and were therefore authenticated

Mycoplasma contamination

All cell line were tested negatively for Mycoplasma

Commonly misidentified lines
(See ICLAC register)

None

Human research participants

Policy information about studies involving human research participants

Population characteristics

Subjects:32
Ethnicity: Chinese 25/32 (78%), Indian 4/32 (13%), Others: 3/32 (9%)
Age, median (range)
41 (23 - 63)
Gender: Female 8/32 (25%), Male 24/32 (75%)
Disease severity: DF 31/32 (97%), DHF 1/32 (3%)
Skin manifestations†
15/32 (47%)
Gastrointestinal symptoms‡
15/32 (47%)
Liver symptoms§
6/32 (19%)
Lowest WBC count, median (range), 109/L
2.7 (1.2 - 8.2)
Highest hematocrit, median, (range), %
45.1 (38.7 - 51.8)
Lowest platelet count, median (range), 109/L
83 (19 - 198)
Highest ALT, median (range), U/L||
49 (17 - 326)
Highest AST, median (range), U/L||
68.5 (29 - 364)

*Cases were classified according to the revised WHO guidelines (44). DF dengue fever, DHF dengue hemorrhagic fever
†Skin manifestations include maculopapular skin rash and/or petechiae
‡Gut manifestations include vomiting, diarrhea and abdominal pain
§Liver manifestations include hepatomegaly and/or rise in liver enzymes of at least 2x upper limit of normal
||ALT and AST levels were not available for 14 of the patient

Recruitment

The patients were diagnosed with acute DENV infection through routine clinical diagnostics (detection of DENV RNA by RT-PCR or NS1 antigen by ELISA). Patients that fulfilled the WHO clinical criteria for acute DENV infection and were positive for immunoglobulin M (IgM) and IgG serology (Panbio Dengue Duo Casette) were also included in the study. Excluded were patients under the age of 18 years, breast-feading, other ski manifestations, and other co-infections, such as HIV

Ethics oversight

The study was approved by by the Singapore National Healthcare Group ethical review board (DSRB 2013/00209 and DSRB 2008/00293).

Note that full information on the approval of the study protocol must also be provided in the manuscript.

Flow Cytometry

Plots

Confirm that:

☒ The axis labels state the marker and fluorochrome used (e.g. CD4-FITC).

☒ The axis scales are clearly visible. Include numbers along axes only for bottom left plot of group (a 'group' is an analysis of identical markers).

☒ All plots are contour plots with outliers or pseudocolor plots.

☒ A numerical value for number of cells or percentage (with statistics) is provided.

Methodology

Sample preparation	Blood was collected in EDTA-treated vacuum tubes at three different time points (acute, post-febrile, and convalescent) after the onset of fever. PBMCs were isolated using Ficoll Hypaque gradient centrifugation and cryopreserved in fetal calf serum (FCS) (Thermo Fisher Scientific) with 10% DMSO (Thermo Fisher Scientific) in liquid nitrogen for later analysis.<br>Suction skin blisters were induced on the forearm of DENV-infected patients using skin suction chambers (Medical Engineering, Royal Free Hospital) and a clinical suction pump. A negative pressure of 25 to 40 kPA was applied to the skin for 2 to 4 hours until a unilocular blister was formed. The blister was covered over night with a rigid adhesive dressing. After 18 to 24 hours, the accumulated fluid inside the blister was aspirated and cells pelleted. After removal of the supernatant, the pellet was resuspended in AIM-V medium (Thermo Fisher Scientific) supplemented with 2% AB human serum, and the cells were analyzed by flow cytometry as described below.
Instrument	Samples were acquired on BD LSR Fortessa equipped with 5 lasers (BD Biosciences). Cytokines were determined by a BioPLex MAGPIX Multiplex reader.
Software	Flow cytometry data analysis was performed using FlowJo version 9.9.4 (TreeStar). Post-processing was performed using SPICE version 5.3 (provided by M. Roederer and J. Nozzi, NIAID, NIH) as well as R version 3.3.1 (The R Foundation of Statistical Computing).
Cell population abundance	No sorting was performed. NK cells account for approximately 10% of lymphocytes and are in blood 90% of the CD56dim NK cell phenotype. The more limited CD56bright NK cells were in some donors less frequent and were analysed if the mother population was at least 50 cells.
Gating strategy	The gating was started looking at time to avoid the sift of fluorescence, followed by doublet exclusion (FSC-H, FSC-A) to only continue with single cells. This was followed by the exclusion of dead cells, CD14 CD19 CD3 and CD4 expressing cells to in the end identify cells expressing CD56 (NK cells). With the help of CD16, they can be divided into CD56bright and CD56dim NK cells.

☒ Tick this box to confirm that a figure exemplifying the gating strategy is provided in the Supplementary Information.
